# Genetic analysis of tolerance to the root lesion nematode *Pratylenchus neglectus* in the legume *Medicago littoralis*

**DOI:** 10.1186/1471-2229-14-100

**Published:** 2014-04-17

**Authors:** Klaus H Oldach, David M Peck, Ramakrishnan M Nair, Maria Sokolova, John Harris, Paul Bogacki, Ross Ballard

**Affiliations:** 1 South Australian Research and Development Institute, Plant Genomics Centre, Waite Campus, Urrbrae, SA 5064, Australia; 2 University of Adelaide, Waite Campus, Urrbrae, SA 5064, Australia; 3 AVRDC - The World Vegetable Center, ICRISAT Campus, Patancheru 502 324, Hyderabad, Andhra Pradesh, India

**Keywords:** Pasture, RLN, Root disease, Gene-based markers, Genetic map, Comparative analysis, Candidate genes

## Abstract

**Background:**

The nematode *Pratylenchus neglectus* has a wide host range and is able to feed on the root systems of cereals, oilseeds, grain and pasture legumes. Under the Mediterranean low rainfall environments of Australia, annual *Medicago* pasture legumes are used in rotation with cereals to fix atmospheric nitrogen and improve soil parameters. Considerable efforts are being made in breeding programs to improve resistance and tolerance to *Pratylenchus neglectus* in the major crops wheat and barley, which makes it vital to develop appropriate selection tools in medics.

**Results:**

A strong source of tolerance to root damage by the root lesion nematode (RLN) *Pratylenchus neglectus* had previously been identified in line RH-1 (strand medic, *M. littoralis*). Using RH-1, we have developed a single seed descent (SSD) population of 138 lines by crossing it to the intolerant cultivar Herald. After inoculation, RLN-associated root damage clearly segregated in the population. Genetic analysis was performed by constructing a genetic map using simple sequence repeat (SSR) and gene-based SNP markers. A highly significant quantitative trait locus (QTL), *QPnTolMl.1*, was identified explaining 49% of the phenotypic variation in the SSD population. All SSRs and gene-based markers in the QTL region were derived from chromosome 1 of the sequenced genome of the closely related species *M. truncatula*. Gene-based markers were validated in advanced breeding lines derived from the RH-1 parent and also a second RLN tolerance source, RH-2 (*M. truncatula* ssp. *tricycla*). Comparative analysis to sequenced legume genomes showed that the physical QTL interval exists as a synteny block in *Lotus japonicus*, common bean, soybean and chickpea. Furthermore, using the sequenced genome information of *M. truncatula*, the QTL interval contains 55 genes out of which five are discussed as potential candidate genes responsible for the mapped tolerance.

**Conclusion:**

The closely linked set of SNP-based PCR markers is directly applicable to select for two different sources of RLN tolerance in breeding programs. Moreover, genome sequence information has allowed proposing candidate genes for further functional analysis and nominates *QPnTolMl.1* as a target locus for RLN tolerance in economically important grain legumes, e.g. chickpea.

## Background

Pasture legumes are used in crop rotations with cereals and oilseeds and bring the benefit of fixing atmospheric nitrogen, improving soil organic matter, soil structure and functioning as a break crop for diseases [1]. Neutral to alkaline soils are widespread in the southern cropping regions of Australia and annual medics (*Medicago* spp.) are widely grown on these soils with *Medicago littoralis* and *M. truncatula* (barrel medic) as the predominant annual medics [2,3]. These conditions appear favourable to the root lesion nematode (RLN) *Pratylenchus neglectus* that is widely distributed in South Australian cropping soils [4] and affects all major legumes and cereal crops [5]. *Pratylenchus* spp. are migratory endoparasitic nematodes that feed and migrate within root cortical tissue causing necrosis and reduced lateral branching of roots upon infection [6]. Damaging the root system, RLN has the potential to reduce water use efficiency, particularly in low rainfall environments [7] and can exacerbate infections by other soil pathogens. Due to its wide host range, resistance and tolerance to RLN has become a breeding priority in wheat and legume breeding as yield losses can be severe. In wheat, losses between 10-30% are attributed to *P. neglectus*, costing the wheat industry in South and Western Australia over $190 M each year [8]. Although being moderately resistant to RLN [4,5], annual medics are intolerant, which means that they are able to inhibit multiplication of nematode numbers but suffer considerable production losses (8-20%) in fields with high nematode numbers [4]. In 2003–2004, the analysis of 389 soil samples from South Australia suggested that over 90% of the soil samples were infested with *P. neglectus* and 27% of the samples contained more than 20 nematodes per gram of soil [4]. Experience based on field assays suggests that each nematode per gram soil translates into one percent of loss in yield in annual medics and severe damage to wheat, especially under low rainfall conditions where nematode-affected root systems are unable to use the sparsely available water [6,9].

The control of RLN through use of nematicides is not a viable option as agrochemicals increase production costs and due to their non-specific side effects represent risks to human health and the wider environment. Chemicals such as dibromochloropropane (DBCP), a common soil fumigant for nematode control, was widely used until the mid 1980s when it was banned after being linked to causing sterility among male workers. Soil steaming is another method to control nematodes but is not practical in broad acre agriculture. Thus, the use of new cultivars that carry genetic resistance and tolerance are the most favoured option to reduce the impact on crop yield due to RLN.

A previous germplasm screen of 225 strand medic lines led to the identification of the *P. neglectus* tolerant strand medic, RH-1 [10]. In order to understand the genetics of the strong nematode tolerance observed in RH-1, we have established a single seed descent (SSD) population of 138 lines segregating for the tolerance trait. Closely linked molecular markers were identified by a dual approach, (1) genetic map construction and (2) QTL analysis and fine mapping using gene-based SNPs. Here, we report on the results of both analyses and the alignment of the genetic information to the fully sequenced *M. truncatula* genome encompassing a physical interval of about fifty genes.

## Results

### Phenotypic analysis of tolerance to *Pratylenchus neglectus*

**Figure 1:**
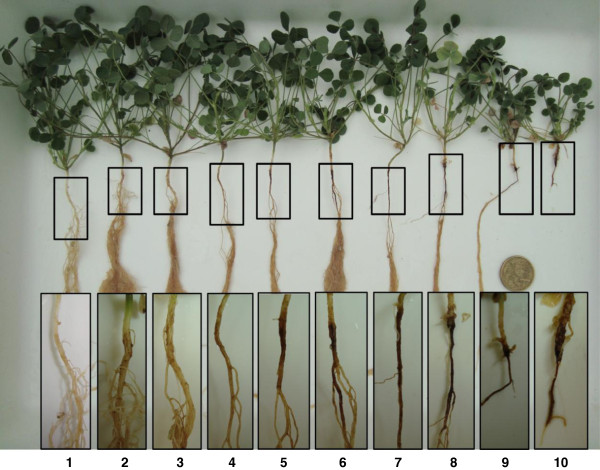
**Scale of root damages observed in the SSD population RH-1 x Herald.** The scoring takes into account lesioning on the upper part of the primary root, increased lesioning and loss of the secondary and tertiary roots, and progression to complete loss of the root in the most intolerant lines. Treatment means differing by more than 1.4 units were significantly different (*P* = 0.05).

Plants that were not inoculated with nematodes had negligible root damage (Score < 0.06) indicating that root damage symptoms observed in inoculated treatments was caused by the nematode. All 138 SSD lines were scored for root damage caused by *P. neglectus* with a broad range of damage observed using a rating scale of 1 (tolerant) to 10 (intolerant) as shown in Figure 1. Highly significant differences (*P* < 0.01) in root damage occurred between the SSD lines. The tolerant (RH-1) and intolerant (Herald) parents responded as expected.

**Figure 2:**
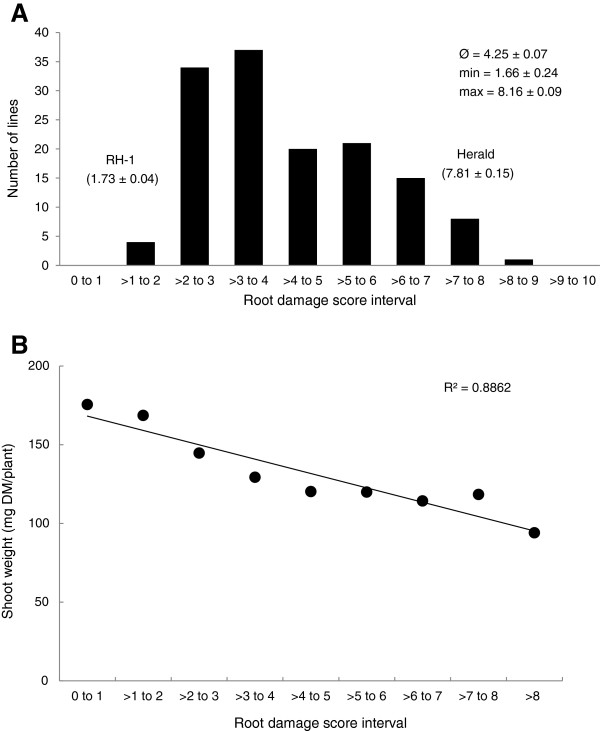
**Phenotypic distribution of root damage scores and their correlation to shoot dry weight in the population RH-1 x Herald after inoculation with *P. neglectus*. (A)** Frequency distribution of mean root damage scores. Presented are mean and standard error values of the RDS in the parents, the population mean (Ø), the minimum (min) and the maximum (max) values are provided. The average LSD (least significant difference) was 1.39. **(B)** High correlation between RDS and shoot dry weight measured using six repeats of each population line.

Tolerant parent RH-1 (score 1.73 ± 0.04) along with three SSD lines had root disease scores less than 2 (Figure 2A). Most commonly, the SSD lines (n = 71) had root damage scores between 2 and 4. The average damage score of the SSD population was 4.24 ± 0.07. The most intolerant SSD line had a root damage score of 8.2 ± 0.09 and was statistically similar to the intolerant parent Herald with score 7.81 ± 0.15 (Figure 2A). The visual RDS was highly correlated to the shoot dry weight (R^2^ = 0.8862) (Figure 2B). The highly significant phenotypic variation between SSD lines, combined with the disparate performance of the parents made the population well suited for use in the subsequent genetic studies. The observed phenotypic distribution in the population follows a bimodal profile describing a quantitative trait controlled by a major and some minor QTL effects.

**Figure 3:**
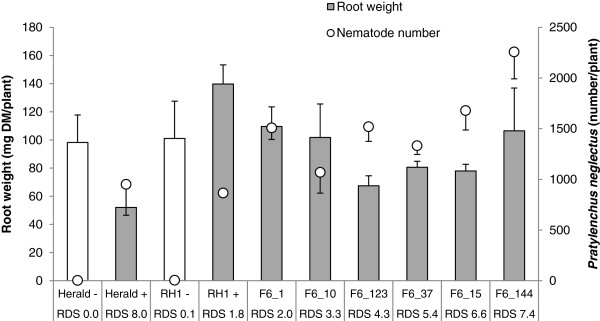
**Quantification of nematode numbers in population parents and SSD lines.** The root dry weight (open bars not inoculated, closed bars inoculated) of Herald, RH-1 and six SSD lines of the Herald x RH-1 cross varying in root damage score (RDS) was measured. The number of *P. neglectus* nematodes was quantified using nematode-specific PCR on roots (open circles). Bars indicate standard errors of four repeats for each plant line.

By definition, tolerance reactions enable a plant to reduce or prevent damage by nematodes while resistance hinders nematodes to multiply [11], which would be noticeable as reduced numbers of nematodes in healthy roots with a low RDS value. To prove that the range of observed root damage in the parents and population lines was due to different levels of tolerance rather than resistance, *P. neglectus* DNA was quantified in the roots of previously scored lines. The quantification of nematode DNA in roots of inoculated plants showed very similar values for both parents, Herald and RH-1 (about 900/plant) (Figure 3). Nematode numbers in the roots of tested SSD lines all exceeded 1000, regardless of RDS score (Figure 3), supporting that the different root damage levels were caused by a tolerance mechanism of *M. littoralis* to *P. neglectus*.

### Genetic map construction and QTL identification

**Figure 4:**
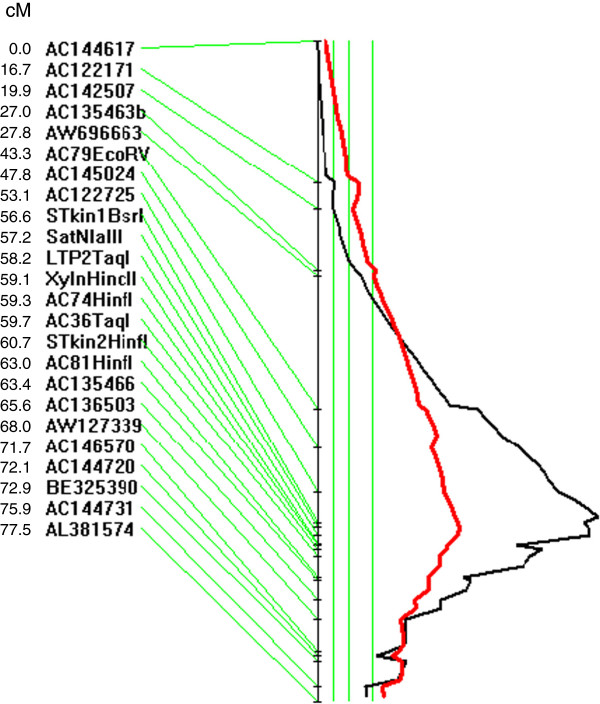
**Genetic likelihood plot of linkage group containing *QPnTolMl.1*in RH-1 x Herald SSD population.** Shown is the identified QTL for tolerance to *Pratylenchus neglectus* in *M. littoralis*, *QPnTolMl.1.* Genetic marker positions are given in cM. Three vertical green lines from left to right indicate likelihood thresholds for suggestive, significant and highly significant QTL, respectively, showing that *QPnTolMl.1* is highly significant. The red line indicates that all marker alleles linked to tolerance are contributed by parent RH-1.

The here developed genetic map of RH-1 x Herald contained 130 markers with 112 *M. truncatula* SSRs and 18 InDel and CAPS markers. The InDel and CAPS markers were derived from polymorphisms identified when sequencing PCR products from RH-1 and Herald using primers that we had designed on published *M. truncatula* BAC sequences at the tolerance locus. A highly significant QTL with a LOD score of 19.9 was identified on a linkage group that contained 30 SSRs and gene-derived markers that, apart from SSR (*AC145024*), were all based on sequence information of *M. truncatula* chromosome 1 (Figure 4). The QTL was called *QPnTolMl.1,* for tolerance to *Pratylenchus neglectus* in *M. littoralis* and explained 49% of the phenotypic variation in the RH-1 x Herald SSD population and the tolerance allele was donated by the tolerant parent RH-1. Information on the genetic linkage and physical position of markers that are closely linked to the QTL is summarised in Table 1. The most closely linked marker was the gene-derived CAPS marker *STkin1BsrI*. The genetic distance between the most closely QTL-flanking gene-derived CAPS markers *STkin1BsrI* and *SatNlaIII* was 0.6 cM in the *M. littoralis* population RH-1 x Herald corresponding to a physical interval of ca. 36 kb. The order of all gene-derived markers on this linkage group reflected the gene order in the sequenced genome of *M. truncatula* (Mt4.0 at http://www.jcvi.org/cgi-bin/medicago/browse.cgi?page=assembly_stats). The physical distance between the genes *Medtr1g071480* and *Medtr1g072420* from which *STkinBsrI* and *XylnHincII* were derived, respectively, spanned 412 kb harbouring 55 genes according to the most recent release (Mt4.0, August 2013).

**Table 1:** **SSR and CAPS markers closely linked to** ***QPnTolMl.1***

**Marker name as in Figure**4	**Primer sequences (5′ – 3′)**	**Amplicon size (genomic)**	**LOD**^**a**^	**% Var**^**b**^
AC122725*	F: CCTGGCACGAGTCATGTAAC	ca. 250 bp	15.8	41
	R: TCACGAGTTTTCAAATTTATCAT			
*STkinBsrI*	F: GTGAAAAGCATGCTGCGGAA	913 bp	19.9	49
	R: AAAGGCAAAGGACCACACCA			
*SatNlaIII*	F: TGCCAACCTCCCTCCAATTC	1072 bp	19.4	48
	R: AAAGGCAAAGGACCACACCA			
*LTP2TaqI*	F: ACGATCACTAGCTTGAGCCC	933 bp	18.7	47
	R: TCTTACTCTGGAAAGCGATTACA			
*XylnHincII*	F: CAGGCCAAAAGCCCTTGTTC	948 bp	18.4	46
	R: CCCAATCGTGTCCCAGTCAA			
*AC74HinfI*	F: GGAGAAACTTGCAGAGCAGGCC	1264 bp	14.1	37
	R: ACGATCCATCAGGAACTCCCGT			

*Accession ID: MtYoungUMinn2006_1_h2_4c11e at cmap.comparative-legumes.org; ^a^Logarithm of odds, ^b^percentage of genetic variation explained by locus.

### Marker validation in advanced breeding lines

The four QTL-flanking markers *STkin1BsrI*, *SatNlaIII*, *XylnHincII* and *STkin2HinfI* (Figure 4) were validated in 9 advanced breeding lines that resulted from phenotypic selection from F_4_ to F_6_ for *P. neglectus* tolerance and other agronomical important traits. The lines carry two different *P.n.* tolerance sources, from RH-1 (*M. littoralis*) or RH-2 (*M. truncatula* ssp. *tricycla*).

Parent RH-2 showed the same tolerance allele as RH-1 for the four tested markers as did all available tolerant breeding lines, 5 *M. littoralis* lines derived from RH-1 x Herald and 4 *M. truncatula* lines originating from RH-2. The matching genotypes between the tolerant *M. littoralis* and tolerant *M. truncatula* ssp. *tricycla* breeding lines suggest that the same tolerance locus to *P. neglectus*, *QPnTolMl.1*, is present in both related species.

### Comparative analysis of QTL *QPnTolMl.1* in *Medicago* and grain legumes

The genomic region of *QPnTolMl.1* in *M. littoralis* had all gene-based markers derived from chromosome 1 of *M. truncatula*. When this region was aligned to the genomes of other legumes using the Legume Information System (LIS, at http://www.comparative-legumes.org), continuous synteny blocks were identified in *Phaseolus vulgaris* (common bean) chromosomes 01 and 07, *Lotus japonicus* chromosome 5, *Cicer arietinum* (chickpea) chromosome 4 and *Glycine max* (soybean) chromosomes 2, 3, 10 and 19. Transcripts of the interval-flanking genes (*Medtr1g071480* and *Medtr1g072420*) and the discussed five candidate genes within the physical interval of the tolerance QTL in *Medicago* were also present in the grain legumes chickpea [12] and soybean, and to a lesser degree in common bean (Additional file 1: Table S1) (http://www.comparative-legumes.org).

## Discussion

The wide distribution and host range of *Pratylenchus neglectus* that includes wheat, canola, chickpea and pasture legumes has made tolerance and resistance target traits in crop breeding programs [4,8,13]. To date, genetic analysis has lead to the identification of five resistance QTL in wheat [14,15] and also in barley [16] but no resistance or tolerance QTL to this pathogen has been described in legumes. To our knowledge, this is the first report on a genetic locus linked to *P. neglectus* tolerance in a legume species. We refer to the observed host response as a tolerance reaction since plants with low root damage scores do not restrict or prevent nematode multiplication, which would be the case for resistance [11].

The distribution of the tolerance phenotypes (root damage scores) in the SSD population showed neither positive nor negative transgressive segregation with both parents as the extreme tolerant or intolerant lines. A single SSD line had a mean RDS of 8.2, which exceeded that of the intolerant parent Herald but the difference was not significant. The genetic analysis of the population revealed the very strong QTL, *QPnTolMl.1*, explaining 49% of the genetic variation. Thanks to the *M. truncatula* genome sequencing efforts ( [17]; Mt4.0 at http://www.jcvi.org/cgi-bin/medicago/browse.cgi?page=assembly_stats) and user-friendly presentation and access to the genome information, a link between genetic and physical map was straight forward.

At the tolerance QTL, the order of the *M. truncatula* SSRs, originally derived from BAC clones, as well as the gene-based markers developed from primers designed on *M. truncatula* gene sequences, matched the marker order in *M. littoralis*. The conserved synteny between these two species of the same genus is expected as it has previously been suggested that *M. littoralis* is a subspecies of *M. truncatula*[18] though now accepted as separate species [19]. *M. littoralis and M. truncatula* can be hybridised [20] and fertile hybrids have been generated to introgress desirable traits from one to the other species and cultivars have been released [21,22]. A sequence comparison of the genomic tolerance region between *M. truncatula* and other legumes suggested that macrosynteny is conserved between medics, *Lotus japonicus* and the grain legumes chickpea, common bean and soybean. In soybean, comparative analysis revealed synteny blocks to four chromosomes due to the two genome duplication events 44 and 15 million years ago [23,24].

Assuming that each gene in the QTL interval flanked by *STkinBsrI* and *XylnHincII* is also present in strand medic, the annotated gene functions (Mt4.0 at http://www.jcvi.org/cgi-bin/medicago/browse.cgi?page=assembly_stats) can be used to screen for candidates that might be responsible for the observed tolerance phenotype. To date, no resistance gene to *P. neglectus* has been cloned in any plant species but a few resistance genes to sedentary nematode species have been isolated. Examples of cloned nematode resistance genes are the *Mi-1.2* gene from tomato conferring resistance to three species of root knot nematodes *Meloidogyne incognita*, *M. arenaria* and *M. javanica*[25]; the *Gpa2* gene in potato mediating resistance to the cyst nematode *Globodera pallida*[26] and the *Hero* gene from tomato conferring resistance to two potato cyst nematodes *Globodera rostochiensis* and *G. pallida*[27]. The structure of the mentioned nematode resistance genes is comparable to those described for viral, fungal or bacterial resistance genes, encoding nucleotide-binding sites (NBS) and leucine-rich-repeat (LRR) domains [28]. Notable is that the reported resistance loci contain clusters of homologous genes that can confer resistance to diseases other than nematodes, e.g. the homologous gene *Rx1* at the *Gpa2* locus mediates resistance to potato virus X [26]. Clusters of 3, 4 and 14 homologous genes within a 52 kb, 115 kb and a 118 kb region were observed for the *Mi*, *Gpa2* and *Hero* locus, respectively [25-27]. In this study, the physical QTL interval with its predicted 55 genes showed no cluster of genes resembling the aforementioned resistance genes or other known resistance gene classes [28]. Given that the observed phenotype is tolerance and not resistance, different types of genes can be expected. The absence of typical R genes might be due to the different mode of action between resistance and tolerance. Gene products that lead to tolerance might have a more direct activity on the invading nematode, e.g. by inhibiting nematode penetration or movement through the root. Tolerance genes do not have to mobilize a defence mechanism that affects reproduction of the nematode in contrast to resistance, as recently observed in the resistance response of wheat to *Pratylenchus thornei*[29]. Tolerance is simply an ability to reduce damage to the plant likely by genes encoding for proteins that can activate or are directly involved in plant defence to pathogens. Whereas no typical *R* genes were present within the tolerance QTL, candidate genes and gene clusters that could play a role in the reduction of damage caused by invading *P. neglectus* were present in the interval. Based on reported characterizations of homologues, the relatively small number of five genes remained to be discussed as candidate tolerance genes. Two genes involved in defence-related signalling were the *patatin-like phospholipase* gene *Medtr1g072190* and the *Rho GTPase* gene (*Medtr1g072280*). Patatin-like phospholipases have been shown to play an active role in establishing successful defence responses against bacterial attacks. For example, silencing the *PLP1* gene in *Capsicum* enhanced susceptibility while overexpression of *CaPLP1* in Arabidopsis improved the plant’s resistance to bacteria [30]. Rho GTPases have been linked to plant defence responses, susceptibility and cytoskeleton organization. Examples of pathosystems where a role for Rho GTPases has been discussed are the rice and the blast fungus *Magnaporthe grisea*[31] and barley against the powdery mildew fungus *Blumeria graminis* f.sp. *hordei*[32]. The three other candidate genes for the improvement of nematode tolerance are the two QTL flanking genes *sat-1* and *xyloglucanase-specific endoglucanase inhibitor*, as well as *lipid transfer protein* genes. The sulfate/bicarbonate/oxalate exchanger and transporter sat-1 could be involved in delivering a higher level of the poisonous oxalate to the site of infection or maintain a higher level in the tolerant roots constitutively. An effective defence against chewing insects due to high levels of calcium oxalate crystals has been described in *Medicago truncatula* where mutant plants deficient in oxalate crystal formation showed clear preference and subsequent severe damage by caterpillar larvae [33]. Both, *xyloglucanase-specific endoglucanase inhibitor* and *lipid transfer protein* genes appeared in small clusters at the tolerance QTL interval. LTPs are secreted proteins and have been proposed to support the transport of hydrophobic monomers like cutin into the cell wall to form protective layers against pathogens [34,35]. A slightly larger gene cluster of three members was found for the gene encoding a xyloglucanase-specific endoglucanase inhibitor, referred to as XEGIP. This protein family and its inhibiting activity on fungal endoglucanases that target xyloglucans in plants had only fairly recently been described [36-38] and a large cluster has been detected in the potato genome [39].

The most closely QTL-linked marker in the SSD population was derived from the gene encoding a Serine/threonine protein kinase Nek2 but Nek2 and related kinases have only ever been reported in the context of cell cycle regulation [40] excluding it as a primary candidate for tolerance to nematodes.

The *in silico* screening for candidate genes conferring tolerance to *P. neglectus* using an advanced genome sequence draft with excellent embedded information has provided a basis for future functional analysis. Candidate re-sequencing in *M. littoralis*, gene expression analysis, transgenesis and a detailed analysis of the infection mechanism, similar to the one described for resistance to *Pratylenchus thornei* in wheat [29] are to follow.

At this stage, practical outputs of this research are four gene-based molecular markers that are available to track the two tolerance sources, RH-1 and RH-2, in *Medicago* breeding programs. The two closely linked markers flanking the *P. neglectus* tolerance QTL can be added to the list of breeder markers previously reported for sulfonylurea tolerance [41] and boron tolerance [42] and help to ensure that the rotation crop *Medicago* is a competitive option in environmentally challenged low rainfall zones. Moreover, with *P. neglectus* causing severe losses in grain legumes like chickpea [43] the identified QTL in *M. littoralis* represents a candidate locus for current genetics research in chickpea.

## Conclusions

In the present study, a genetic mapping approach led to the identification of a major QTL for tolerance to the root lesion nematode *Pratylenchus neglectus* in *Medicago littoralis*. SSR and gene-based CAPS markers at the QTL interval mapped physically to chromosome 1 in *M. truncatula* and comparative genome analysis suggested that the region also exists as synteny blocks in other sequenced legume genomes. Thus, the identified QTL is a candidate locus for RLN tolerance in pasture and grain legume species. Supportive of this idea is that the linked gene-based markers derived from *M. littoralis* can also be used to select RLN tolerance derived from *M. truncatula* ssp*. tricycla*. Moreover, five genes that are predicted in the corresponding *M. truncatula* genome interval are likely candidates involved in reducing the root damage caused by nematodes.

## Methods

### Plant material

A cross between the two strand medic (*M. littoralis*) genotypes RH-1 (tolerant) and Herald (intolerant) was made in 2004 to develop a single seed descent (SSD) population with a final number of 138 lines that were used for genetic analysis. The population was specifically developed by SARDI’s pasture breeding group to investigate the observed strong tolerance phenotype of line RH-1 (see below) to *Pratylenchus neglectus*. The SSD lines were at stage F_5:6_ when we analysed tolerance phenotypically and extracted DNA for genetic analysis.

In addition, 9 advanced breeding lines with quantified *P. neglectus* tolerance levels were used for genotyping with four PCR markers spanning the tolerance QTL. Advanced breeding lines are defined as largely homozygous breeding lines (F_6_) that combine different desirable traits and are potential future cultivars. Here, 9 advanced breeding lines were derived either from additional RLN tolerant lines of the mapping population, RH-1 x Herald, or from crosses between RH-2 and different *M. truncatula* cultivars. Herald was bred to generate a strand medic cultivar with resistance to the pests spotted alfalfa aphid and bluegreen aphid [44]. RH-1 is a wild accession collected from Cyprus in 1987 and classified as strand medic (*M. littoralis*) whilst the tolerant line RH-2, also a wild accession that was collected from Cyprus in 1983 has been classified as a barrel medic subspecies, *M. truncatula* ssp*. tricycla*. The advanced breeding lines were derived from crosses between the RH-2 and *P. neglectus* intolerant progeny from crosses between the barrel medics Caliph, Jemalong and the strand medic Angel. The progeny were repeatedly phenotyped, tolerant lines were kept and the best five lines based on repeated agronomical performance in growth room and field were included in the linkage analysis between markers and *P. neglectus* tolerance.

### Plant growth conditions

Seeds from lines for nematode testing were surface sterilised in 80% ethanol for 15 s, followed by 3% hypochlorite solution for 3 min and then thoroughly rinsed with sterile water. Seeds were stored at 4°C on moist filter paper in petri plates for 2 days and then placed in a 25°C incubator for 16 hrs to germinate.

Germinated seeds were planted into a mixture of approximately 200 g of coarse sand and vermiculite (50:50 vol:vol) contained in square plastic pots (50 × 50 mm diameter × 120 mm depth) and moistened with 40 mL of nutrient solution [45] containing a small amount of N (6 ug N/mL as CaNO_3_). Plants were grown for 44 days in a controlled environment room maintained at 20/15°C day/night temperature and 14/10 hr light/dark regime. Plants were watered with reverse osmosis water as required and additional N supplied (8 mL per pot of 19 mM CaNO_3_.4H_2_0) at 18, 25, 32 and 38 days after sowing.

### Nematodes

*Pratylenchus neglectus* was obtained from the SARDI population #99 (originally sourced from a soil collected from Cambrai, South Australia in 1991) maintained on carrot callus as previously described for *P. vulnus*[46]. Nematodes were collected by placing carrot callus in funnels in a misting chamber under intermittent aqueous mist of 10 s every 10 min for 96 h [47] at room temperature (22°C). Nematodes extracted were counted and diluted with water to the inoculum concentration of 3000 nematodes per 1 mL. Nematodes were applied to each seedling in two inoculations, at 4 and 10 days after sowing. At each inoculation, 3000 nematodes were applied as two 500 μL aliquots, each dispensed into a 25-mm-deep hole in the soil mix on either side of the seedling, formed by gently pushing a 1000 μL Gilson disposable pipette tip into the soil. Nil nematode treatments received two 500 μL aliquots of water only. After inoculation, holes on either side of each seedling were filled using a small amount of the surface soil in each pot.

### Phenotypic assessment of *P. neglectus* tolerance

The tolerance response of the 138 SSD lines to *P. neglectus* was assessed in a growth chamber experiment. Eight repeats (each pot containing one seedling) of the 138 SSD lines and 32 repeats of each parental line (RH-1 and Herald) were inoculated with nematodes. An additional 8–16 repeats of each parental line were not inoculated with nematodes (negative controls). After the second nematode inoculation (10 days after sowing) all pots were arranged in a randomised block design. Repeats from each treatment were distributed and randomised within eight blocks in the growth chamber. Thirty-two days after the second inoculation, plants were removed from the pots and roots washed free of sand. Individual root systems were scored visually on a scale from 0 to 10 with low scores indicating low root damage (or high nematode tolerance) and *vice versa* (Figure 1). Differences between population individuals were assessed using one-way analysis of variance (ANOVA).

The nine validation lines resulted from the same phenotypic screen as used for the SSD population applied on progeny that was derived from crosses between the two tolerance sources, RH-1 and RH-2. Both sources were used to introgress tolerance into agronomically important varieties. Plants at F_4_, F_5_ and F_6_ were repeatedly assessed for tolerance and only tolerant plants (RDS ≤ 4) kept for variety development.

All roots of the inoculated mapping population lines were dried at 60°C. Nematode tolerance was tested by quantifying *P. neglectus* DNA in the roots of the parental and selected SSD lines that had been scored with different levels of tolerance. Total root DNA was extracted by the SARDI Root Testing Service [48-50] and the amount of *P. neglectus* DNA was quantified using a real-time TaqMan PCR system with primers specific to the internal transcribed spacer (ITS) region of *P. neglectus* (unpublished data). Nematode DNA was quantified and used with a standard curve that was established with known amounts of DNA per nematodes. The quantified levels of nematodes in the roots were expressed as number of nematodes per plant (Figure 3).

### Genetic map construction

For map construction, microsatellite markers were obtained from primer sequence information at the Medicago HapMap project website (http://www.medicagohapmap.org/) and the list of reported markers [51,52]. Out of the 240 screened SSRs, 110 showed a polymorphism between the parental lines RH-1 and Herald and were subsequently used for genotyping of the entire SSD population. These SSRs were used to construct a genetic linkage map using MapManager QTXb20 [53] with the Kosambi mapping function [54] and a linkage criterion of *P* = 10^-4^. The marker order was finalized using RECORD [55]. After QTL identification, 18 additional markers were developed on the basis of BAC sequences and later on using genes at the critical QTL region.

SSR markers were assayed in a 9.5 μL reaction mixture containing 0.2 mM dNTP, 1x PCR buffer, 1.5 mM MgCl_2_, 10 μM each of forward and reverse primer, and 2.5 U Taq DNA polymerase (Qiagen). A touchdown profile was used for PCR cycling comprising an initial denaturation step of 94°C for 2 min, followed by a total of 37 cycles of 94°C for 30 s, an annealing step for 30 s and 72°C for 30 s. Initial annealing temperature was 59°C and was reduced by 0.5°C for each of the next 8 cycles. The remaining 29 cycles had an annealing temperature of 55°C, the program ended with a 5 min extension at 72°C. The SSR-marker-amplified products were separated on 8% non-denaturing polyacrylamide gels (Sigma Aldrich, Australia) at constant 200 V for 180 min, and visualized with ethidium bromide staining.

### Development and use of DNA markers

Additional markers were developed after QTL analysis based on the genome sequence of *M. truncatula* (Mt3.5). The sequence of co-located BACs and annotated genes therein were used to design primers for further SNP identification and development of CAPS (cleaved amplified polymorphism) markers. An additional 18 markers, 3 based on InDels and 15 being CAPS, were added to the genetic map. PCR reactions were run at 94°C for 2 min, followed by 94°C for 30 s, 59°C for 30–60 s and 72°C for 60 s. Amplicons were either directly separated on a 2% agarose gel (InDels) or first digested by a SNP specific restriction enzyme (as indicated in the marker name in Table 1) according to the manufacturer’s protocol. Marker names, primer sequences and amplicon sizes of the mapped loci close to *QPnTolMl.1* (Figure 4) are provided in Table 1.

### Comparative analysis

To compare the QTL region of *QPnTolMl.1* among different legumes genomes, the positions of predicted genes in *M. truncatula* were aligned with corresponding regions in common bean (*Phaseolus vulgaris*), soybean (*Glycine max*), and chickpea (*Cicer arietinum*) (Additional file 1: Table S1). Databases used for comparisons were *Medicago truncatula* genome sequence release version 4.0 (jcvi.org/medicago/jbrowse), Legume Information System, LIS (medtr.comparative-legumes.org/gb2/gbrowse/Mt3.5.1) and Gramene release 39 (gramene.org), the latter two databases using *M. truncatula* genome sequence release version 3.5.

## Abbreviations

RLN: Root lesion nematode; QTL: Quantitative trait locus; SSD: Single Seed Descent; M.: *Medicago*; P.: *Pratylenchus*; SSR marker: Simple sequence repeat marker.

## Competing interests

The authors declare that they have no competing interests.

## Authors’ contribution

KHO conceptualised the idea, carried out all genomics and bioinformatics part of marker development and drafted the manuscript. RB was involved in conceptualising the idea, carried out all the phenotypic analysis and helped progressing the SSD population. DMP derived the breeding lines and developed the SSD population from the initial cross by RMN who was also involved in the conceptualisation of the project. MS carried out all of the SSR and gene-based mapping supported by breeding line genotyping by JH with PB having carried out the polymorphism checks of the SSR markers. All authors read and approved the final manuscript.

## Supplementary Material

Additional file 1: Table S1 Comparative analysis of physical position of QTL *QPnTolMl.1* (flanking genes) and discussed candidates (bold font) in *M. truncatula* and grain legumes according to LIS. Click here for file
