# Supplementary material for: Genetic analysis of tolerance to the root lesion nematode Pratylenchus neglectus in the legume Medicago littoralis
Source: BMC Plant Biol. 2014 Apr 17;14:100. doi: 10.1186/1471-2229-14-100 (PMC4021308; doi:10.1186/1471-2229-14-100)
Supplement: Additional file 1: Table S1 — Comparative analysis of physical position of QTL QPnTolMl.1 (flanking genes) and discussed candidates (bold font) in M. truncatula and grain legumes according to LIS. [file 1471-2229-14-100-S1.doc]

**Additional file 1**

**Table S1.** Comparative analysis of physical position of QTL *QPnTolMl.1* (flanking genes) and discussed candidates (bold font) in *M. truncatula* and grain legumes according to LIS

| Gene locus in *M. truncatula* genome (Mt3.5) | Functional annotation of gene product (Mt3.5) | Physical position in *M. truncatula* (Mt3.5) | Matching transcript in *Cicer arietinum* | Matching transcript in *Glycine max* | Matching transcript in *Phaseolus vulgaris* |
| --- | --- | --- | --- | --- | --- |
| *Medtr1g071480* | Serine/threonine protein kinase Nek2 | Mt1:17644457..17649131 | Cicar_201201_TA003782 | - | - |
| *Medtr1g071530* | **Sulfate/bicarbonate/oxalate exchanger and transporter sat-1** | Mt1:17673157..17680402 | Cicar_201201_TA018888 | TA61948_3847 | AF527434 |
| *Medtr1g071720* | Non-specific lipid-transfer protein | Mt1:17783238..17785753 | Cicar_201201_TA032155 | TA64208_3847 | - |
| *Medtr1g071730* | **Non-specific lipid-transfer protein** | Mt1:17786661..17787810 | - | - | - |
| *Medtr1g072190* | **Patatin-16** | Mt1:18026345..18028166 | Cicar_201201_TA003247 | TA61886_3847 | CV531538 |
| *Medtr1g072280* | **Mitochondrial Rho GTPase 1** | Mt1:18063342..18071899 | Cicar_201201_TA007634 | CX548148 | - |
| *Medtr1g072380* | Xyloglucan-specific endoglucanase inhibitor protein | Mt1:18112532..18114639 | Cicar_201201_TA001298 | TA50332_3847 | - |
| *Medtr1g072420* | **Xyloglucan-specific endoglucanase inhibitor protein** | Mt1:18124541..18126393 | Cicar_201201_TA001298 | BF009805 | - |
| *Medtr1g072450* | Xyloglucan-specific endoglucanase inhibitor protein | Mt1:18134176..18135791 | - | BQ629877 | - |

Note: *Medtr1g07380* and -*450* are annotated as *Basic 7S globulin* genes in Mt3.5 but as *xyloglucan-specific endoglucanase inhibitor* genes in Mt4.0 as Basic 7S globulins are xyloglucan-specific endoglucanase inhibitor protein.
